# Supplementary material for: Interactions between attributions and beliefs at trial-by-trial level: Evidence from a novel computer game task
Source: PLoS Comput Biol. 2022 Sep 26;18(9):e1009920. doi: 10.1371/journal.pcbi.1009920 (PMC9536582; doi:10.1371/journal.pcbi.1009920)
Supplement: S1 Appendix — (DOCX) [file pcbi.1009920.s001.docx]

**S1 Appendix. Staircase procedure** We used a double staircase procedure, with the stair used for each trial chosen randomly and updated based on the outcome as described below. Each stair was characterised by three variables:

- the overall size of the maze, *n*: if the maze is conceived as a square *n*X*n* matrix of “maze chambers”, with each chamber having four possible walls, which can be present or absent, all the combinations of wall patterns that form a valid maze give the total set of available mazes for a given maze size *n*; there are four possible “levels” of maze size on our staircases, consisting in two values for *n*, one of which is randomly drawn, with equal probability, before generating the maze on each trial: level 0 has available *n* values {3, 5}, level 1 has available *n* values {5, 5} level 2 has available *n* values {5, 7} and level 3 has available *n* values {7, 7}.
- the average frequency of maze rotations during a trial ν: all trials start in the normal upright position and the first rotation, resulting in a randomly chosen orientation at an angle of 90, 180 or 270 degrees with respect to the upright one, happens 30 frames (1.5 seconds) later; a random number is then uniformly drawn from the interval (ν − 10, ν + 10), representing the number of frames until the next rotation; the angle of the rotation is drawn randomly with equal probability from the three available options (90, 180, 270 degrees) every time a rotation happens. The available values for ν were between 20 and 140 frames, and a staircase step was 10 frames.
- the available time for the trial, *t*: the available values for *t* are 10, 12 and 14 seconds, with the staircase step being 2 seconds.

Both staircases started with the same value for the available time, the maximum one of 14 seconds. One of the staircases started on level 1 of the maze dimensions available and on ν = 30 frames, the other started on level 2 of the maze dimensions available and on ν = 80 frames. These values were updated as follows: if still possible, ν was increased (decreased) by 10 for wins and losses respectively; when the upper (lower) limit was reached, the maze dimension level was increased(decreased), if still possible; when the upper (lower) limit was reached for this as well, the available time *t* was decreased (increased) if possible.
