# Supplementary material for: Interactions between attributions and beliefs at trial-by-trial level: Evidence from a novel computer game task
Source: PLoS Comput Biol. 2022 Sep 26;18(9):e1009920. doi: 10.1371/journal.pcbi.1009920 (PMC9536582; doi:10.1371/journal.pcbi.1009920)
Supplement: S2 Appendix — (DOCX) [file pcbi.1009920.s002.docx]

**S2 Appendix. Empirical difficulty and skill** We attempted to extract objective measures of both difficulty and skill from our data by first establishing a measure of difficulty, and then defining skill with respect to it. Intuitively, a difficult trial is one that a randomly picked subject in the population is likely to lose, while an easy trial is one that subjects are likely to win; subjects’ skill is measured with respect to this difficulty - the more likely subjects are to win difficult trials, the more skilled they are considered to be. The staircase controls several objective dimensions along which trials vary, and which could contribute to their objective difficulty. We did not know a priori how these individual factors might contribute to an integrated difficulty score, instead, we attempted to infer the identities and weights of the different factors from data, assuming that difficulty predicts trial outcome. Specifically, given a set of objectively measurable task aspects that might act as factors in determining difficulty, *f1,f2,..fk* , we assumed that there is a stable set of weights, *w1,w2,..,wk*, representing the contribution that they each have towards determining the outcome, such that on any trial *t*

$$\begin{matrix} p(o(t)=1;\mathbf{w}) & =\sigma(\mathbf{w}_{d}^{T}\mathbf{f}_{d}(t)),\text{where} \\ \mathbf{w}_{d}^{T} & =(w_{0},w_{1},...w_{k}) \\ \mathbf{f}_{d}^{T}(t) & =(1,f_{1}(t),...f_{k}(t)) \\ f_{i}(t) & =\text{the measured value of factor}f_{i}\text{at trial t} \\ o(t) & =\text{outcome at trial t, and} \\ \sigma(x) & =\frac{1}{1+e^{-x}}\text{, the sigmoid function.} \end{matrix}$$

Given ***w****d*, difficulty for trial *t* can be computed as *d(t) = −****w****dT* ***f****d(t)*. In order to obtain, for each subject, an objective, external difficulty measure, not influenced by their own skill, we inferred ***w****d* separately for each subject, by predicting outcomes for all remaining subjects (note that here we present data from a pilot population of participants). We fitted all outcomes from all subjects with logistic regression models with several combinations of regressors and their interactions and, based on the cross-validation score, pickled the model which best explained outcomes across subjects. This included length of correct path to maze exit, proportion of non UP orientations, path length×orientation interaction, time available and necessary minimum speed as features. S1 Fig, shows a summary and sanity checks of the resulting empirical difficulty measure.

Analysis of the accuracy of difficulty as a predictor of outcome suggests that the staircase might have failed to track subjects’ skill levels: outcome prediction based only on difficulty values, according to the simple model *p(o(t) = 1) = σ(−d(t))*, is highly accurate, ranging from 0.69 to 0.91, with an average of 0.84 and s.d. of 0.04 across subjects(see S2 Fig). Tracking the subject’s skill level would explore difficulty ranges where difficulty alone is insufficient as a predictor. The fact that difficulty alone is such a good predictor of outcomes implies that little room is left for skill; this is indeed what we found in our analyses aimed at defining skill, to which we turn next. We modelled objective skill as the evolving factor that intermediates between the objective difficulty of a trial and success, and attempted to extract an objective measure of skill from data by extending the previous difficulty-only model for outcomes:

$$\begin{matrix} p(o(t)=1;\mathbf{w}_{p}) & =\sigma(\mathbf{w}_{p}^{T}\mathbf{f}_{p}(t)+\mathbf{w}_{d}(t)),\text{where} \\ o(t) & =\text{outcome of trial t} \\ \mathbf{f}_{p}(t) & =\text{vector of performance regressors at trial t} \\ d(t) & =\text{difficulty at trial t} \\ \mathbf{w}_{p} & =\text{performance weights, parameters} \\ \mathbf{w}_{d} & =\text{difficulty weight, fixed} \end{matrix}$$

For a given value of ***w****d*, fitting the above model to trials from one subject produces performance weights ***w****p*; these can then be used to obtain the trial by trial skill measure, computed as the performance contribution to the outcome prediction *s(t) =* ***w****p****f****p(t)*. We used three performance features, computed on a trial-by-trial basis, namely the proportion of pauses, the proportion of correct key presses, and the proportion of wrong key presses that would have been correct in the normal UP orientation. We compared the model’s prediction accuracy for a range of negative values for ***w****d*, as well as for ***w****d = 0*, which is equivalent to using only performance features to predict outcomes ( see S2 Fig); we also compared these accuracies with that obtained when using difficulty as the only predictor of outcomes. Note that the accuracy of the models including performance features is a training accuracy (and therefore likely an overestimation), as these models were fitted on the same individual subject data on which accuracy was computed; this is not the case for the difficulty-only model.

As illustrated in S2 Fig, this comparison showed that difficulty alone is overall more predictive of outcome than performance features alone, and that adding performance features to the difficulty-only model only marginally improves overall accuracy. Computing outcome prediction accuracy as a function of difficulty level provides a more detailed account of the models’ performance, showing that there is only a narrow range of difficulty values for which difficulty alone fails to predict outcome, and where including performance features significantly improves prediction accuracy.
