# Supplementary figures and images for: Interactions between attributions and beliefs at trial-by-trial level: Evidence from a novel computer game task
Source: PLoS Comput Biol. 2022 Sep 26;18(9):e1009920. doi: 10.1371/journal.pcbi.1009920 (PMC9536582; doi:10.1371/journal.pcbi.1009920)

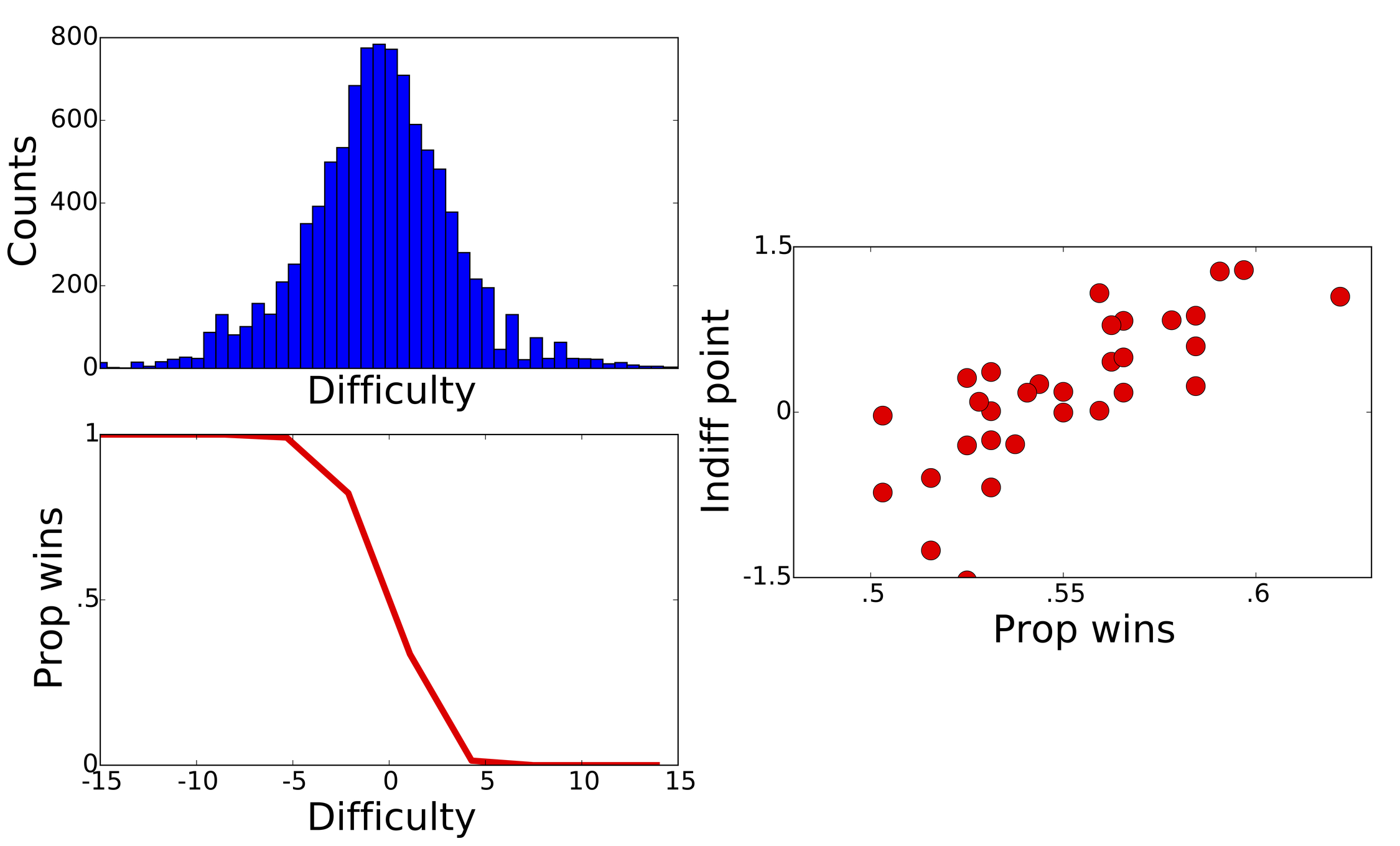

Supplement: S1 Fig — Left: data pooled from all subjects; top: distribution of difficulty values; bottom: relationship between difficulty and the proportion of wins. Right: relationship between difficulty indifference point—difficulty value for which subject is equally likely to win or lose the trial—and the proportion of trials won out of all trials; each dot represents a subject; r2 = 0.6, p-value = 3 * 10−7. (TIF) [file pcbi.1009920.s003.tif]

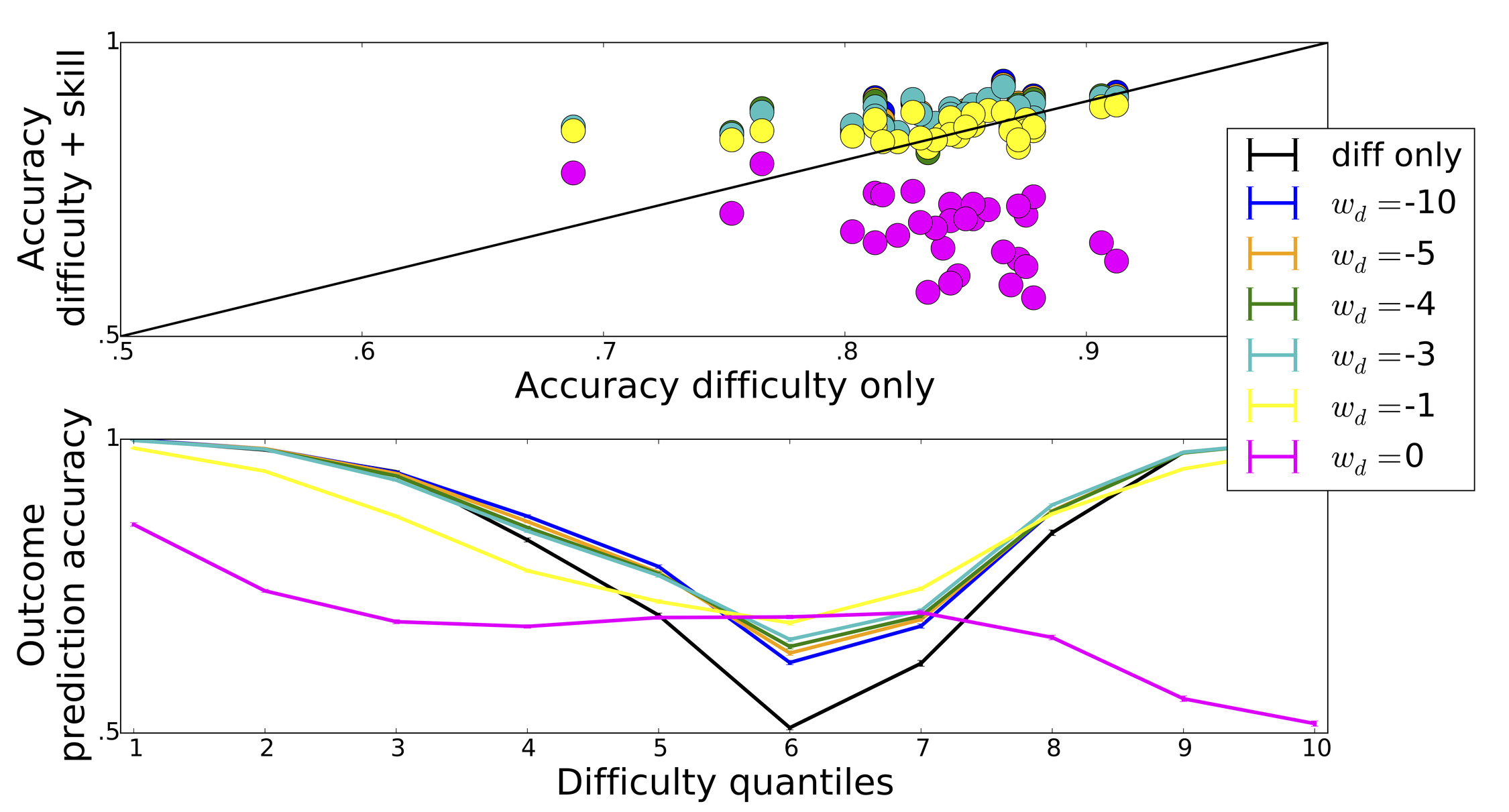

Supplement: S2 Fig — Colours correspond to different wd values in the skill and difficulty models; note that wd = 0 (purple) is equivalent to a model with skill only; black is used for the model with difficulty only. Top: overall accuracy; each dot represents one subject. Bottom: accuracy per difficulty level; mean ± s.e.m across subjects; difficulty was z-scored for each subject and discretised in 10-quantiles. (TIF) [file pcbi.1009920.s004.tif]

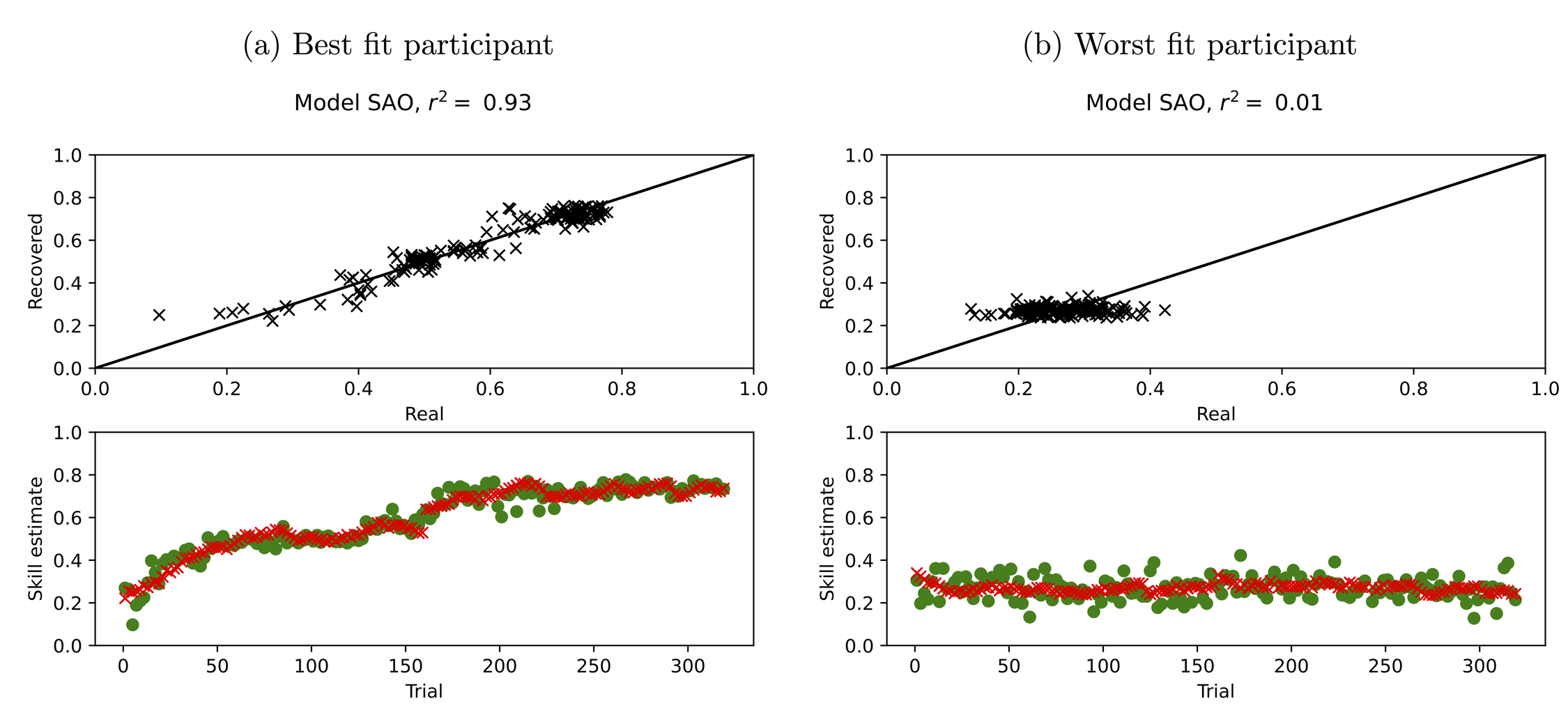

Supplement: S3 Fig — Example best (A) and worst (B) fit participants, SAO model of skill estimates, self condition. Top: participant responses vs underlying skill recovered with mean posterior parameters. Bottom: time series of participant responses and underlying skill recovered with mean posterior parameters. (TIF) [file pcbi.1009920.s005.tif]

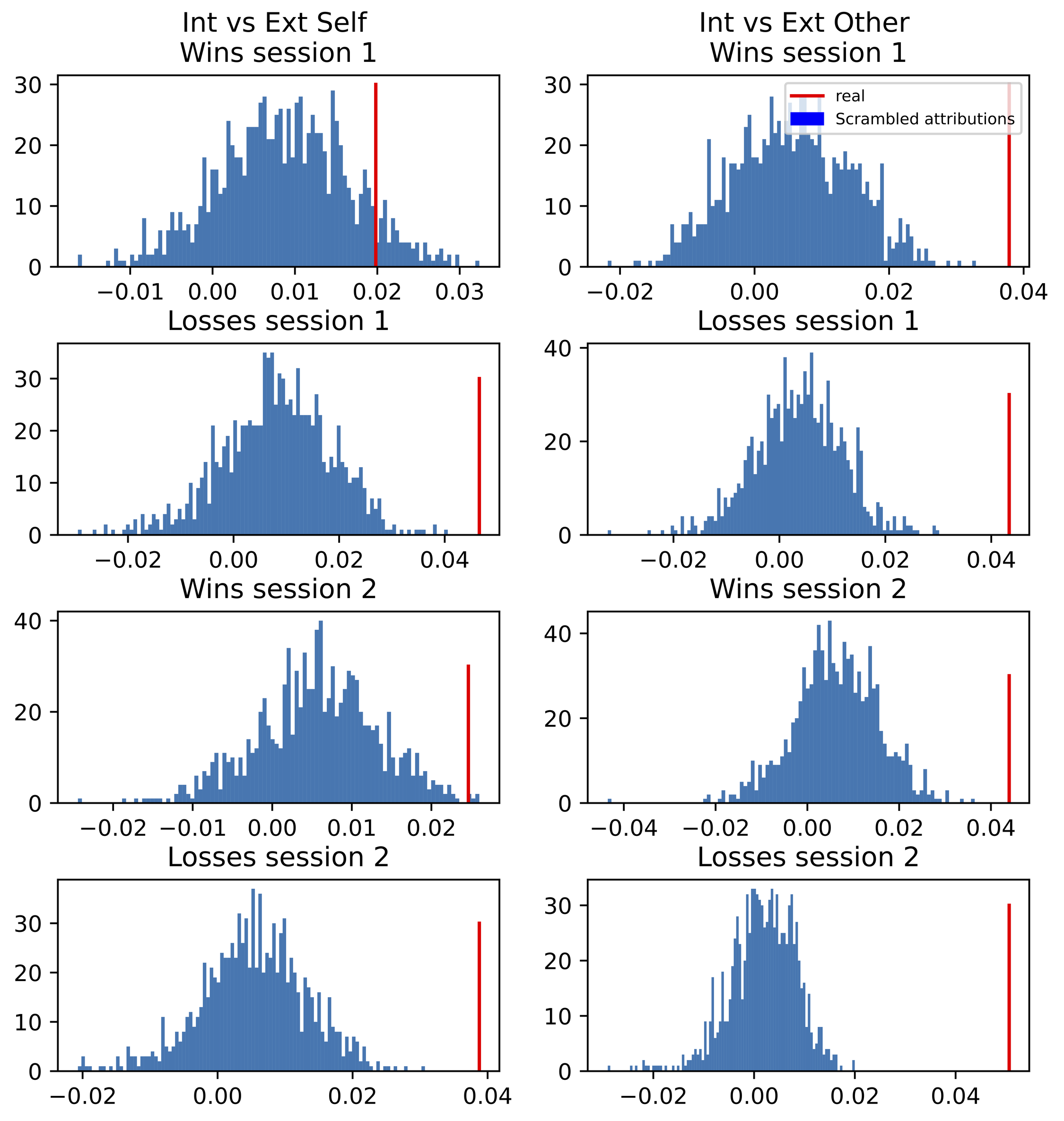

Supplement: S4 Fig — To test whether the attribution effects observed in model agnostic analyses were detectable in model parameters we refitted the model to data with scrambled attributions and compared the observed differences in learning rates to the ones obtained in the real data. Attribution response shuffling: effect on difference between learning rates of the SAO model. Analyses were performed as follows: for each of 1000 permutations, attribution responses of each individual participant were shuffled and the SAO model was refitted. For each combination of outcome and session, the difference between the corresponding internal and external α parameters was averaged across participants. The resulting shuffle distribution is compared with the average difference obtained from fitting the real data. Left: self, right: other. (TIF) [file pcbi.1009920.s006.tif]

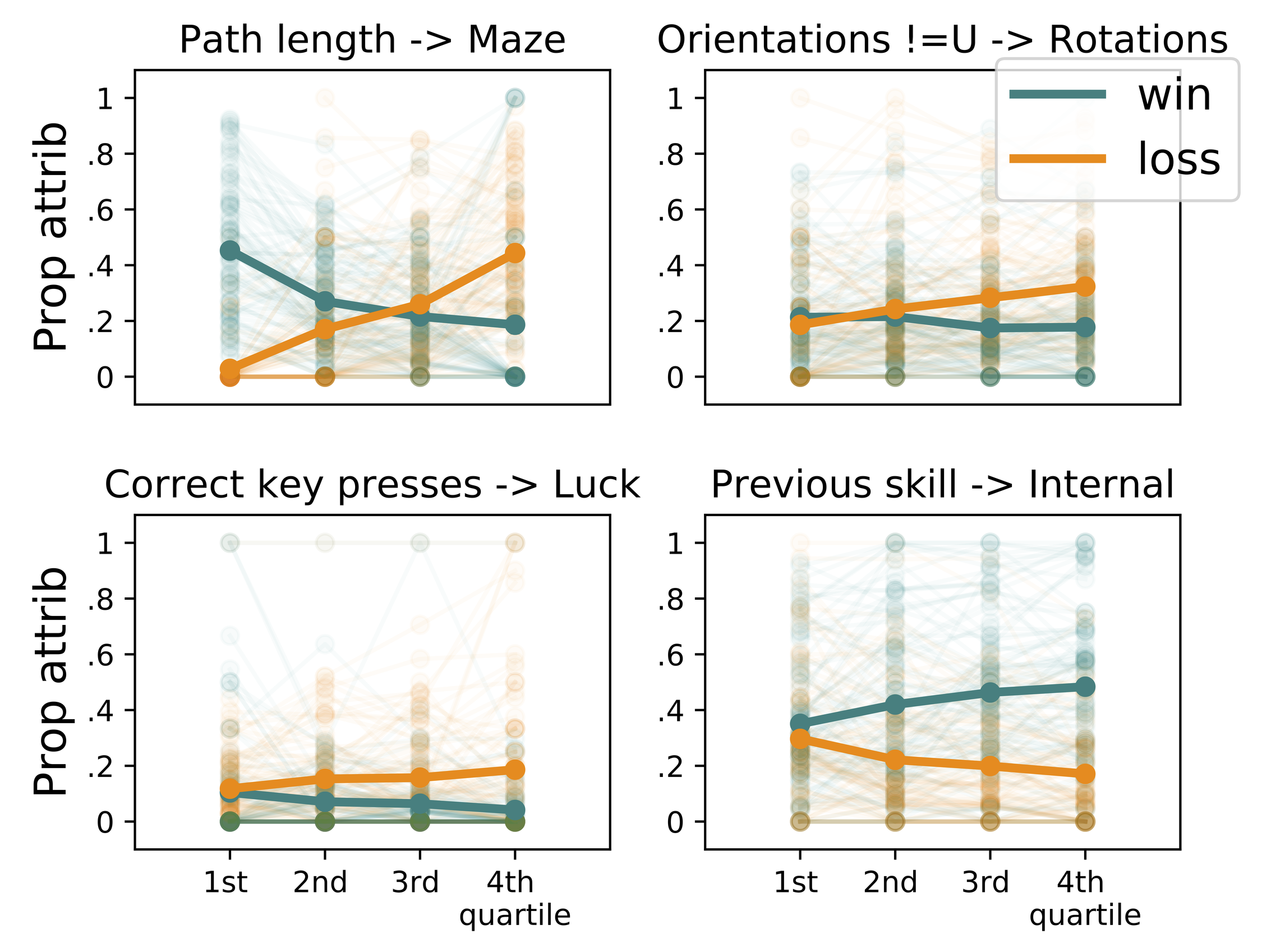

Supplement: S5 Fig — Features of interest and attributions summary other. Faded lines represents individual participants, bold lines represent mean ± s.e.m across participants. Orange: losses, teal: wins. (TIF) [file pcbi.1009920.s007.tif]
