# Supplementary material for: Interactions between attributions and beliefs at trial-by-trial level: Evidence from a novel computer game task
Source: PLoS Comput Biol. 2022 Sep 26;18(9):e1009920. doi: 10.1371/journal.pcbi.1009920 (PMC9536582; doi:10.1371/journal.pcbi.1009920)
Supplement: S1 Table — (DOCX) [file pcbi.1009920.s008.docx]

|  | Self | | Other | |
| --- | --- | --- | --- | --- |
|  | Session 1 | Session 2 | Session 1 | Session 2 |
| Win | t=2.41  p=0.0056  d=0.22 | t=3.67  p=0.0002  d=0.28 | t=4.45  p<1/5000  d=0.5 | t=6.64  p<1/5000  d=0.69 |
| Loss | t=5.88  p<1/5000  d=0.36 | t=5.57  p<1/5000  d=0.36 | t=5.98  p<1/5000  d=0.69 | t=7.64  p<1/5000  d=0/84 |

To test whether the attribution effects observed in model agnostic analyses were detectable in model parameters we performed permutation tests on mean posterior parameters from the winning model. Permutation test results and effect sizes, comparisons between learning rates for internal vs external attributions, conditioned on outcome and session, SAO model. See Fig 7 in main text.
