# Supplementary material for: Interactions between attributions and beliefs at trial-by-trial level: Evidence from a novel computer game task
Source: PLoS Comput Biol. 2022 Sep 26;18(9):e1009920. doi: 10.1371/journal.pcbi.1009920 (PMC9536582; doi:10.1371/journal.pcbi.1009920)
Supplement: S2 Table — (DOCX) [file pcbi.1009920.s009.docx]

|  | Path length | | | |
| --- | --- | --- | --- | --- |
|  | Self | | Other | |
|  | Win | Loss | Win | Loss |
| Internal | m=0.11  p<1/5000 | m=-0.03  p<1/5000 | m=0.14  p<1/5000 | m=-0.04  p<1/5000 |
| Maze | m=-0.15  p<1/5000 | m=0.12  p<1/5000 | m=-0.14  p<1/5000 | m=0.12  p<1/5000 |

To test wether feature effects extracted from the parameters of the winning models are significantly different from 0 we performed sign permutation tests on the mean across participants.
