# Supplementary material for: Interactions between attributions and beliefs at trial-by-trial level: Evidence from a novel computer game task
Source: PLoS Comput Biol. 2022 Sep 26;18(9):e1009920. doi: 10.1371/journal.pcbi.1009920 (PMC9536582; doi:10.1371/journal.pcbi.1009920)
Supplement: Attachment — Submitted filename: response_to_reviewer_comments.pdf [file pcbi.1009920.s013.pdf]

1 Reviewer 1

In this paper, Zamfir & Dayan use a novel sensorimotor skill learning task to test a computational model that posits a dynamic, cyclical relationship between outcome attributions (i.e., to the self or to external perturbations) and one's belief about their abilities in a task. Specifically, their model predicts that individuals will produce greater updates to their belief about their skill after attributing an outcome internally; moreover, as skill improves over time (or rather, one's belief about it), one will also be more likely to attribute positive outcomes internally, and negative outcomes externally. Using simulations, the authors show how their straightforward model can generate these effects. Then, using a novel navigational button-pressing task, they show that people's task outcome attributions and ongoing skill judgments follow the predicted trends. Taken together, the results support their formalization of the so-called "attribution-self representation cycle theory."

Overall this is a strong paper. The authors propose a novel formalization of an important idea, and introduce an impressive task to test their computational hypotheses. The modeling framework is rather straightforward and clear, and the simulation, fitting, and behavioral results generally provide strong support for the stated conclusions. I have several critiques concerning design and interpretation, highlighted below.

Thank you very much for these kind comments and for the suggestions.

Comment 1 I may have missed something, but the implementation of the attribution and skill judgments seems to have a potential design flaw - the latter always followed the former during the task. It is possible that this could lead to a demand effect, where making a particular type of attribution biases one's subsequent skill judgment. (This, in effect, might confound the results in the predicted direction I believe?) At the very least this potential drawback should be discussed, though perhaps additional analyses (or experiments with counterbalanced judgment orderings) could be implemented to directly address it.

Response to comment 1 This is a critical point that we had indeed considered in the design of the task and its analyses. Our choice of the ordering of the attribution and skill questions following every trial leads to a time series of alternating attribution and skill belief responses which, in connection with our analyses, is intended to reduce potential influences of demand characteristic biases on any measured effect. The ASRC postulates continuous reciprocal influences between beliefs and attributions, which are conceived as alternating time series, i.e., attributions and belief updating constantly both follow and precede each other. For implementation purposes we needed to discretize the sampling of these variables, probing attributions and beliefs on a trial-by-trial basis. While we cannot completely exclude the possibility that these prompts might have influenced participants' responses, our design reduces the likelihood of such effects contaminating our results. We chose the ordering of the attribution and skill questions following every trial so as to obtain a time series of alternating attributions (A) and skill belief (S) responses ($A^0, S^0, A^1, S^1 ... A^T, S^T$) which reduces influences of demand characteristic biases on the effects of interest in our analyses: the effect of attribution was measured on skill belief *updates* (i.e., the effect of A^t on $S^t - S^{t-1}$), rather than on the immediately following skill belief report itself (S^t); conversely, the effect of skill was measured on the attribution collected after the following trial (i.e., the effect of S^t on A^{t+1}). Under the alternative question ordering we would have obtained the alternative time series, $S^0, A^0, S^1, A^1, ... S^T, A^T$, and measuring the effect of skill belief on attributions would have involved the effect of $S^t on A^t$, which, as an absolute rather than comparative measure, would likely have been more prone to corruption by demand

characteristics. In response to this comment, we made several changes to the paper, in order to explain our choice of the ordering, draw attention to the potential influences of the questions on measured effects, and underline how our analyses are designed to reduce such influences on our results.

The changes are as follows:

- in the experiment design section: Our choice of the ordering of these questions following every trial leads to a time series of alternating attribution and skill belief responses which, in connection with our analyses, is intended to reduce potential influences of demand characteristic biases on any measured effect (see discussion in 3.2 below).

- in the section dedicated to analyses of skill estimates: Note that these effects are detected on the way that skill beliefs are updated rather than their actual level. This is intended to reduce potential contributions of demand characteristics arising from the fact that participants provide attributions just before reporting their skill. We cannot, however, entirely rule out demand characteristics.

- in the part of the discussion containing reflections on the task: The ASRC postulates continuous reciprocal influences between beliefs and attributions, which are conceived as alternating time series, i.e., attributions and belief updating constantly both follow and precede each other. For implementation purposes we needed to discretize the sampling of these variables, probing attributions and beliefs on a trial-by-trial basis. While we cannot completely exclude the possibility that these prompts might have influenced participants' responses, our design reduces the likelihood of such effects contaminating our results. We chose the ordering of the attribution and skill questions following every trial so as to obtain a time series of alternating attributions (A) and skill belief (S) responses ($A^0, S^0, A^1, S^1...A^T, S^T$) which reduces influences of demand characteristic biases on the effects of interest in our analyses: the effect of attribution was measured on skill belief *updates* (i.e., the effect of A^t on $S^t - S^{t-1}$), rather than on the immediately following skill belief report itself (S^t); conversely, the effect of skill was measured on the attribution collected after the following trial (i.e., the effect of S^t on A^{t+1}). Under the alternative question ordering we would have obtained the alternative time series, $S^0, A^0, S^1, A^1, ...S^T, A^T$, and measuring the effect of skill belief on attributions would have involved the effect of $S^t on A^t$, which, as an absolute rather than comparative measure, would likely have been more prone to corruption by demand characteristics.

Comment 2 Throughout the text, "skill" itself (objective performance) and "skill belief" are often conflated. This delineation could be made more explicit - what can we learn about discrepancies between these two constructs? Moreover, the fact that including both the objective and subjective skill metrics appears to be important for modeling attributions (if I followed the second fitting analysis correctly) is an interesting result that could be more thoroughly discussed.

Response to comment 2 This is a very important point. Note that objective performance cannot be precisely equated with objective skill, because the trials differ in difficulty. However, since there is not an objective measure of difficulty, we did not define, measure, discuss or analyse objective skill in this paper. In model-agnostic and model-dependent analyses of attribution responses, the distinction is thus between momentary performance and participants' reported beliefs about skill. In response to this comment we now explicitly specify throughout the text the meaning of "skill" to which we are referring and we added to the Discussion section a brief discussion of the several important distinctions to be made regarding the notion of skill.

The changes are as follows:

In all our analyses of participants' data, "skill" always referred to participants' own skill estimates. There are, however, several important distinctions concerning skill: the timescale distinction between skill and momentary performance: a skilled player might still make mistakes and hence produce poor momentary performance; and distinctions between objective skill and perceived skill and the subtler issue of actually defining objective skill in relationship with objective difficulty: a skilled player is one who can prevail in difficult circumstances, while both skilled and less skilled players can win easy trials. Due to the nature of the task and the lack of an unambiguous quantification of difficulty, we did not define, measure, discuss or analyse objective skill in this paper. This also precluded us from investigating relationships between objective and perceived skill, and relationships between any such discrepancies and attributions. Using an externally validated measure of difficulty together with a precisely calibrated staircase would allow participants' true underlying skill level to be closely tracked and objectively measured, enabling investigations into relationships between the accuracy of participants' beliefs about skill and their patterns of outcome attributions, and also their scores on questionnaire-based psychological measures. Various other issues that we were not able to address could be examined such as whether participants display self-serving biases in their skill estimates, and, if so, whether such biases are associated with self-serving attribution patterns or with higher self-esteem scores.

Comment 3 The decision to include the "Other" condition is somewhat under-motivated. While there was at least one interesting result from that condition (the valence effects), how it is situated in the border manuscript felt a bit confusing. What were the explicit hypotheses about this condition?

Response to comment 3 The attribution-self-representation cycle (ASRC) theory only refers to self. However, the well-established actor-observer effect (Jones and Nisbett 1987, Malle 2006) suggests that there will be a systematic bias in attribution about the self versus the other. Thus, it seemed compelling to explore how biases about the self might work when others are involved. Given that the ASRC had itself not previously been operationalized at a fine timescale, we hesitated to formulate prior hypotheses about how our results would be modulated by the self/other distinction.

We made changes to the Experimental design section to make this explicit: Note that the ASRC only refers to the self. There is, however, ample research on distinctions between self and other within the attribution literature, notably the actor-observer effect (Jones and Nisbett, 1987; see Malle, 2006, for a review) which implies that there might be a bias in attributions for the self versus another. We introduced the "other" condition to explore whether aspects of this bias would impact our task.

Comment 4 The number of presses (accuracy) is used as the main performance metric. I think it could be useful to analyze, or at least mention, changes in response (or completion) time over different conditions and amounts of training. Or perhaps a "speed-accuracy tradeoff" metric could be computed? Might this represent a more direct measure of skill in this type of speeded sensorimotor task?

Response to comment 4 The main performance metric indeed involved the proportion of correct key presses. Time constraints are present and essential in our task, but the core difficulty of the task is introduced by maze rotations, i. e. changes in relationships between key pressed and movement on the screen. The metrics the reviewer suggests are certainly interesting in general; however, they are not applicable here because of the task conditions – for instance,

keeping the same key pressed after a rotation (i.e., a 0ms reaction time) is actually the correct response.

Little things: -Significant versus non-significant correlations should be made clear in figure 9
-Typo “cuuld” on page 3
-In the figures, “Skill” might be changed to “Skill belief” or something similar

Thank you for these observations, we have made these corrections to the figures.

2 Reviewer 2

In this work, causal attributions were tested in online participants using an original skill game. Participants' beliefs about their skills and causal attributions of experienced outcomes were collected trial by trial. The authors found that participants updated their skills beliefs more after internally than externally attributed outcomes; they also found that participants gave themselves more credit for wins and less blame for losses as their skills increased. Interestingly, these patterns correlated with questionnaire-based measures of self-esteem, locus of control and attributional style.

I particularly liked the computational part of the paper: the authors designed an elegant model in which the update of one's skill belief is determined by a learning rate whose value depends on both the valence of the outcome and the participant's attribution (internal/external), allowing for fine-grained (trial by trial) tracking of potential interactions between skill beliefs and attributions. Model comparison revealed that allowing learning rates to differ for internal and external attributions improves the model evidence; both model-dependent and model-agnostic analyses showed a significant effect of attribution on skill estimates (internally attributed outcomes =¿ stronger effects on beliefs about skills). I was less impressed with the correlations between the questionnaire-based measures and the individual parameters of the winning model, but given the usual low predictability of these questionnaires, the results presented are nonetheless far from negligible.

Thank you very much for your generous comments and helpful suggestions.

I have a few comments about the interpretation/discussion of the results:

Comment 1. I find the functional interpretation of your results a bit short, and particularly focused on personal-level dispositions (self-esteem, well-being, etc.). I think the (brief) discussion you make of your results would benefit from a discussion of other work close to yours, such as the studies conducted on learning asymmetries in the RL domain (e.g. Dorfman et al. 2019, Psych Sci, on self-serving bias in causal inference under benevolent vs adversarial conditions, or Chambon et al. 2021, Nat Hum Beh, on learning asymmetries in controllable vs uncontrollable environments, and perhaps also Cockburn et al. 2014, Neuron). These studies do not focus on skill sensitivity as in the present study, but rather on sensitivity to the controllability of the current situation, which somehow echoes low and high skill situations. Interestingly, most of these results found a higher learning rate for positive outcomes, which is in stark contrast to what the you found here. I think this should be discussed further in the paper, especially since these authors suggest that a higher sensitivity to positive outcomes is not only a matter of maintaining a positive view of oneself (e.g. promoting self-esteem and well-being), but actually resolves a problem of credit assignment (e.g. Cockburn et al.) and/or allows for extra-reinforcement of actions that meet internal needs (e.g. Chambon et al.).

Response to comment 1 Thank you for raising this issue. We have amplified the discussion of the functional interpretation of our results.

Thank you in particular for encouraging us to consider these particular sets of studies. Note first, though, that higher learning rates for negative prediction errors have also been found even in rather similar contexts to these (Gershman 2015, Niv et al 2012) – so there are relevant precedents.

We appreciate the reviewer's suggestion to consider the relationship between controllability and

skill. However, we think that 2-armed bandit problems, in which participants learn to choose between two sources of reward, while very useful for examining aspects of prediction and outcome learning, are not so compelling as ways of investigating relationships between causal attributions and learning about oneself (or other, in the other condition). Conversely, since the way that outcomes arise depends so critically on detailed aspects of performance in our task (rather than a simple choice), the sequence of prediction errors is far from under experimental control, making it hard to compare aspects such as differential learning rates. It is also important to mention that learning rates in the two cases are of different nature: they reflect changes in preference for different choices in the bandit case, and changes in reported beliefs about skill in our case.

To come to the specific studies that you mention:

Dorfman et al's main result involves discounted learning when outcomes are attributed not to the reward source, but to external intervention. To the extent that our task can be compared with Dorfman et al's, this result is consistent with our observation of smaller learning rates for externally attributed outcomes: in both cases, this can be interpreted as participants reasonably learning less from outcomes they deem less relevant. Dorfman et al also find higher learning rates for positive than for negative outcomes, however, as argued, comparison between this result and ours is not straightforward, as in Dorfman et al's case learning rates are derived from preferences for different reward sources, while in our case, they are derived from evolving beliefs about performance.

Chambon et al's main result indicates stronger learning from positive vs from negative experienced outcomes, with the opposite pattern in learning from counterfactual outcomes in the context of free choice. This asymmetry is consistent with a choice-confirmation bias; however as learning rates in this case refer to changes in preferences between options, this result is not straightforward to translate for the case of learning rates derived from modelling evolving beliefs about the self, which is the case in our task. How such confirmation biases relate to motivations that are at play when learning not about reward sources, but about one's performance, as is the case in our task, is indeed an interesting question for future work.

Cockburn et al's study investigates mechanisms underlying people's preference for options they have freely chosen in the past over equally valuable options which they have not. The study shows evidence for an involvement of dopaminergic striatal plasticity, a by-product of mechanisms which ensure credit assignment to the agent's actions leading to reward. This is an interesting result relating neural circuits with a RL component and behavioural preference. Credit assignment mechanisms and causal attributions are fundamentally related, however there are crucial differences between this study and ours, making a direct comparison between results tricky. The mechanisms involved in boosting individual rewarded motor actions such as the ones involved in the 2-armed bandit task employed by Cockburn et al are likely very different from mechanisms involved in estimating the extent of one's responsibility for an outcome in the complex game setting we employ in our study.

In response to this comment we made changes to the Discussion section, mentioning the papers the reviewer suggested and their relationship with our work. The changes are as follows:

Selective attendance to, or overweighting of, positive information has been found in studies investigating self-evaluation or the processing of other self-relevant information (Lyons et al., 2020; Sharot et al., 2011) (see Blaine and Crocker (1993); Campbell and Sedikides (1999); Mezulis et al. (2004) for reviews), where presumably it reflects biases toward maintaining a positive view of oneself, as well as in a large number of RL studies (Caz e and Van Der Meer, 2013; Cox et al., 2015; Frank et al., 2007; Lefebvre et al., 2017; Wrase et al., 2007; Yacubian et

al., 2006) (though the opposite pattern has also been reported, see Gershman (2015); Niv et al. (2012)). Note that significant differences are likely to exist between both credit assignment and motivation mechanisms in conventional forms of RL (such as two-armed bandit tasks) vs self-evaluation tasks such as ours, and that learning rates in the two cases are of different natures: they reflect changes in preference for different choices in the former, and changes in self-related beliefs in the latter.

Comment 2. Heightened attention to negative feedback can be essential for survival in harsh environments, and might be particularly relevant in learning contexts where it can be used to improve performance" : this claim is actually controversial at best (see Gershman 2015, PBR, or Chambon et al. 2021, NHB, for lack of learning rate adaptation as a function of the harshness of the environment), and contradicts optimality analyses from Cazé and van der Meer 2013 (cited in the present paper) which suggest that it is more advantageous to have an positivity bias in a harsh and poorly rewarded environment - which makes sense since positive outcomes are rarer in such environments and therefore more informative. Also, given that your task does not vary the harshness of the environment, or, say, the amount of reward available in distinct blocks (e.g. "poor" and "rich" blocks), I find it difficult to conclude anything about the valence asymmetry found here (negative LR > positive LR).

Response to comment 2 Thank you for this point. It is quite true that our experiment was not designed for the purpose of testing the effect of environment harshness and is not really suitable for such a test (not the least because of the staircase); we therefore do not aim to make any claims about this factor. We do think it worth pointing out that our observation, which is consistent with similar work on learning about one's own performance, suggests such a test would be worth performing in future work.

To elaborate, unlike Gershman, Chambon and Caze (noting that empirical results from Gershman are not consistent with simulation results from Caze), our study does not involve learning about the values of two reward choices whilst choosing between them, but learning about one's performance in a more elaborate and engaging task, while having to perform extended sets of actions. Furthermore, the learning rates in the two cases are of a different nature, reflecting changes in preference for different choices in the bandit case, versus changes in reported beliefs about skill in ours. Additionally, outcome valence is modelled as different learning rates based on experienced outcome in our case, while it is modelled as different learning rates for positive vs negative prediction errors in the above papers. While modelling environment harshness as amount of available reward is suitable for 2-armed bandits context, it is not the natural interpretation of harshness in the context of our task, where it might more adequately be modelled as task difficulty (which was operationally controlled via the staircase) or effort requirements (which are harder to assess). In complex scenarios involving extended sequences of actions and therefore multiple aspects of performance, learning about and improving aspects of one's performance is both crucial and challenging, in marked contrast with 2-armed bandit-like scenarios of the type used in these studies.

To reflect the reviewer's concern, we made changes to the related Discussion section to clarify our observation:

Heightened attention to negative feedback might be particularly relevant in complex environments requiring extended sequences of actions, as such scenarios involve multiple aspects of performance, making learning about and improving aspects of one's performance both crucial and challenging (Maier and Seligman, 2016; Muller-Pinzler et al., 2091). Our experiment was not designed for the purpose of testing the effect of environment demands on learning rate adapt-

ability in learning contexts and is not suitable for such a test, but our observations suggest this would be an interesting goal for future work.

Comment 3 Since psychiatric disorders are mentioned in the paper: it may be important to recall that abnormal control beliefs/causal inferences are not of one type and may vary along a continuum from the experience of increased internal control (e.g. delusion of omnipotence) to little or no control (e.g. delusion of control). Thus, it might be relevant to specify in the paper how the parameters of the model (either competence level sensitivity, attribution sensitivity, or learning rate asymmetries) can be abnormally tuned to predict which end of this continuum (from delusion of omnipotence to delusion of control) the patient is on. With respect to depression, your hypothesis of a "disproportionate effect of attributions for particularly significant events" is interesting but seems at odds with so-called "depressive realism" where, precisely, depression is explained by the absence of bias in attribution styles (no valence-induced asymmetries - i.e. no abnormal weighing of positive or negative events) or, more parsimoniously, by the patient's passivity (depressed patients produce fewer actions than control participants, and are therefore less likely to make illusory causal attributions, see Matute et al., 2015, Front in Psychology).

Response to comment 3 Thank you for raising this important point. Although we do mention psychiatric disorders in the paper, and indeed embarked on this study in the light of theories of dysfunctional cognition, we considered it critical to start by establishing (and refining) the task in a population of healthy volunteers. We did think it important to provide some hints as to possible dysfunctional modes in the ASRC via the simulations, which show example parameter combinations that can lead to vulnerable behavioural patterns, but a full analysis of psychiatric disorders would require us to administer the task to a clinical population and to look at fitted parameters. This is a very compelling direction for future work.

Future simulation analyses could also be brought to bear on the issue of how negative biases relate to depressive realism (Alloy and Abramson, 1979, Martin et al., 1984; Vaźquez, 1987) , which is certainly an issue that requires additional investigations. Indeed, as the reviewer will doubtless be aware, the extent of depressive realism is somewhat controversial, as various studies have failed to find associations between depressive symptoms and higher accuracy in contingency perception, or have found only partially consistent results (Alloy et al., 1981; Alloy and Abramson, 1982; Benassi and Mahler, 1985; Presson and Benassi, 2003) (and Allan et al., 2007; Moore and Fresco, 2012, for reviews). Many studies did not use clinically depressed subjects, but rather subjects labeled as such based on BDI indexes; studies involving depressed patients challenged the depressive realism view, providing evidence that depressed patients have negative biases in their judgements which are not incompatible with them appearing more accurate in some cases (Carson et al., 2010; Kaney and Bentall, 1992), and that they share with non-depressed subjects the illusion of control bias - a tendency to estimate they have some degree of control when none is present (Venkatesh et al., 2018; Carson et al., 2010; Moore and Fresco, 2012; Va zquez, 1987; Presson and Benassi, 2003; Kaney and Bentall, 1992 .We do not in the present study have patient data that would be necessary to contribute to this debate other than by speculation.

Minor: The paper does not seem to make a (strong) distinction between attribution style and locus of control (the latter being even used as a measure for the former). I think treating them as interchangeable constructs is questionable – and indeed debated in the literature. Locus measures expectations (or anticipations) about the future, while attributional style measures explanations about past outcomes.

Response to minor We are sorry if we gave the wrong impression. Our main results refer not to attributional style, but to participants' own individual attributions for individual trials, elicited

with questions explicitly asking for a main cause of the past outcome. We therefore intend there to be no conflation with the locus of control. In analyses relating behaviour in our task with questionnaire measures, relationships between participants' attributions for individual trials and their attributional style are evaluated using the Attributional Style Questionnaire rather than the Locus of Control questionnaire. We also evaluate relationships between participants' behaviour and their responses on the Locus of control questionnaire, however this is a separate analysis, which keeps the ASQ and the LC questionnaires separate.

3 Reviewer 3

The authors adapt classic reinforcement learning models to capture how agents update beliefs about their skills after receiving wins or losses. Thereby, the authors offer a new computational perspective on the long-debated question of how humans attribute wins and losses to internal vs. external causes. Overall, I find the article interesting and sound (i.e., the new models, the task, the analyses, and the cited literature). Apart from one suggestion regarding the relation between skill and outcomes, my main concern is that the presentation of the model, the results, and their implications is often rather convoluted. Please find my specific suggestions below.

Thank you for this generous view and for your suggestions, which have been useful in improving the paper.

Comment 1 Objective relation between skill (or difficulty) and outcomes: The authors mention this point in the discussion (in 3.2) but in my view this is too late and not completely justified. When reading the formulas for the skill belief updates (in 2.1), I immediately wondered how the model captures that actual skill is usually related to outcomes. Depending on the task, this relationship can vary, i.e., the probability of a win given a certain level of skill depends on how controllable the outcome is (e.g., in some tasks the outcome is completely determined by skill; in other task a positive outcome is still rather unlikely even at the highest level of skill). Crucially, agents can have undue assumptions about (i) their level of skill (which the newly introduced model captures) and/or about (ii) how much their skill determines the outcome.

a. I am wondering if the latter point could be formalized as $p(o|s)$ and varied in the simulations of the model. Agents could form (and update) beliefs about this probability, which could then influence how they attribute, i.e., the $p(a|o, s)$. Thereby, one could simulate tasks with different skill-outcome-relationships.

b. Even in the empirical data, $p(o|s)$ could be obtained from the step-wise procedure that was used to adjust the difficulty of the employed task.

c. Even if such further analyses not possible in the current settings (as the authors imply in the discussion), it would have been really helpful for me to read early-on about these limitations in the scope of the model.

Response to comment 1 The outcome dependence on skill is indeed not explicitly modelled in our simulations, although it is implicit in the skill updates. The scope of the simulations in the present work was limited to establishing a formal scaffolding for the empirical work and to illustrating various behaviours that the system can produce, even in quite simplified settings. In particular, we did not, in the simulations, explore the space of participants' underlying model of the relationship between skill and outcomes, because identifying such a model in our data is not something our task was designed to allow. Expanding the simulations to include various models of this relationship and or assumptions about control is a very interesting avenue for future work, and we are grateful for the useful suggestions in a).

Determining $p(o|s)$ from our empirical data, as suggested in b) is unfortunately currently not possible, as we do not have a validated way of determining the skill level, and we do not have a validated way of measuring difficulty. While, in principle, skill could be determined from the staircase procedure, this is complicated by the fact that the staircase is controlled by a number of interacting components, and does not allow a straightforward uni-dimensional reading of skill, as well as by the fact that the staircase steps are most likely too coarse, as indicated by results of

additional analyses reported in the supplementary material (see Figure 2). Obtaining objective measures of difficulty and skill and exploring the nature of participants' internal skill models remain goals for future work.

In response to c) we have made changes to the simulations section of the paper, to explicitly mention these limitations earlier. Changes are as follows:

Note that this simple model for skill updates implicitly captures the assumption that high skill is likely to be associated with wins, and low skill with losses, but it does not explicitly encode participant's assumptions about environment controllability, or about the relationship between their ability and outcomes, as our simulations are not aimed at investigating these assumptions (and our behavioural task does not assess this formally.

Comment 2. Presentation: Suggestions for more clarity. I'll start with two suggestions that are more relevant in terms of content. After that, I have more nitty-gritty suggestions.

a. Simulations: I like that the authors first show simulations of their model and vary parameters. However, the motivation for varying specific components is not clearly given.

Response As noted above, the main intent of the paper was to propose and test the behavioural task; the much more limited intent of the simulations was to put the ASRC on the sort of formal footing that the task could hope to illuminate. Furthermore, since we did not test a range of clinical populations, we were not expecting to be in a good position to examine a psychiatrically-relevant parameter range even for simulations that more precisely aped the behavioural task.

Often the authors mention verbally which "component" (e.g., "reciprocal influences," "vulnerability," etc.) is varied but they do not explicitly say to which parameter this component is linked (instead the readers needs to look into the precise values in the brackets).

Response Sorry. We now explicitly state which is the relevant parameter for the model components mentioned.

I had the impression that the authors want to show that initial conditions do not matter that much. So, many simulations are motivated by such very important checks. However, in my view many simulations with varying parameters could be motivated by showing how the model can capture different strategies in diverse populations. I think that overall a lot more could be done to illustrate how simulated agents with different learning rates (which capture levels of self-esteem, etc.) would learn. I am deliberately a bit vague here but I am convinced that the authors could show more simulations that give a better picture of what to expect from "simulated psychiatric patients."

Response As noted, the scope of simulations in the present work was quite limited. Using simulations to explore behaviour patterns produced by a range of parameters, and by interactions between them, or to include the effect of different strategies in different populations remains an important goal, and these are very useful suggestions for future work.

b. Higher learning rate for internal attributions after losses (Figure 7b): Seeing this, I first had the impression that participants were not self-serving. The authors discuss this point also in relation to the study by Müller-Pinzler et al. 2019). To understand this crucial point better, it would be helpful to show that the number of these trials is rather small because participants themselves make the "attribution decision." For me, this result nicely exemplifies why it is so important to look at the interaction of outcomes and attributions. Therefore, it could be

explained in more detail.

Response to comment 2b Thank you for this perceptive point. There is indeed an apparently contradictory pattern: in the "self" condition, participants display self serving attributions, but have higher learning rates for negative outcomes than for positive ones. It is true that internally attributed losses are fewer than externally attributed ones, but note that the outcome asymmetry in learning rates is present for both internally- and externally-attributed outcomes; thus it is not the case that the number of trials involved is small, participants generally learn more from negative outcomes than from positive ones in the "self" condition. While the attributional bias is also present in the "other condition", the asymmetry in learning rates does not seem to be present there. This suggests that learning from negative outcomes might be particularly important when one can improve one's behaviour in a learning context (self, but not other, similar to Müller-Pinzler et al.). The fact that the asymmetry between learning rates for losses and learning rates for wins is larger for internally attributed outcomes (i.e. those for which participants believe themselves to be most responsible), is consistent with this.

c Title: "evidence from a novel task" This wording in the title is rather unspecific. Also, the wording "high temporal resolution" suggest an M/EEG study or some study using a specific measurement; maybe "trial-by-trial" or "step-by-step" would be better.

Response to Comment 2c Thank you for this suggestion. We changed the title to "Interactions between attributions and beliefs at trial-by-trial level: Evidence from a novel computer game task", to make it more specific.

d Abstract and introduction: I completely understand that the authors aim to apply their approach for understanding psychiatric disorders. But they often set the undue expectation that they will report data from patients. In the abstract, for example, it is unclear if the authors also assessed clinical scores. The "computational psychiatry aspect" could simply be mentioned as an outlook for future studies.

Response to comment 2d Sorry for being misleading. We now state already in the abstract that the participants were healthy volunteers.

e Abstract: "Substantial population of online participants:" The authors could directly mention the sample size in the abstract.

Response to comment 2e Due to drop-out between sessions, the number of participants for the two conditions is unfortunately different. Unfortunately, this makes the description of the number of participants a bit unwieldy for the abstract.

f Introduction: The authors provide a really nice and detailed example on the fundamental attribution error. The authors refer to the substantial and long-established literature on attributions. There's one bit that I didn't quite get: How do the biases relate to the homeostatic regime?

Response to comment 2f Indeed attribution has historically been studied with a focus on biases and unhealthy regimes, due to the interest of these topics for psychiatry. From the ASRC theory perspective, these biases can coexist with a homeostatic regime, as long as they do not push each other in catastrophic vicious cycles, or other aberrant dynamics (Bentall 2003). Understanding how the various biases interact when maintaining the healthy regime is of course a fundamental goal. In this work our aim was merely to make a first step in this direction, by establishing the existence of event-by-event mutual interactions between attributions and

beliefs,as postulated by the ASRC.

g Relationship with questionnaires: Here, the authors list some specific hypotheses but do not motivate them properly.

Response to comment 2g Our analyses of relationships between questionnaire scores and behaviour were exploratory and therefore based on intuitive hypotheses about which aspects of behaviour in our task would be related to the trait-like characteristics measured by questionnaires. In response to this comment we made changes to the Relationships with questionnaires section, as follows:

We first investigated relationships between questionnaire measures and aspects of behaviour in our task that seem intuitively related to them. We hypothesized that self esteem scores would be related to patterns of self assessment in both skill and attribution responses: specifically, we expected participants with higher self-esteem scores to judge themselves as more skilled, to show larger differences in skill estimate updates after wins vs after losses, and to show larger differences between the proportions of internal attri- butions for wins vs losses, compared to participants with lower self-esteem. We also investigated the relationship between internal locus of control and the proportion of internal attributions, and that between scores on the internality positive and internality negative subscales of the ASQ and the corresponding proportions of internal attributions in our task

h Figures: i. Please do not use "See text for details"
ii. The y-axes is missing for Figures 8c and 8d.
iii. The models in Figure 7c have to be spelled out (and explained) in the caption.
iv. Often it is unclear how the data were split. E.g., quartiles in Figure 8a: Quartiles on what?
v. Figures 5a and 6c: These numbers add up to 1, don't they? So, some forms of cumulative plots would be easier to grasp.

Response: Thank you for this careful reading. We have fixed these issues, and have examined the figures carefully.
